# Depression and HIV risks: Engaging older African American women in HIV prevention education through the church

**DOI:** 10.3389/frph.2023.898032

**Published:** 2023-02-14

**Authors:** Megan T. Ebor, Aurora P. Jackson

**Affiliations:** ^1^ San Diego State University, San Diego, CA, United States; ^2^ School of Social Work, College of Health and Human Services, San Diego State University, San Diego, CA, United States; ^3^ Department of Social Welfare, Luskin School of Public Affairs, University of California, Los Angeles, Los Angeles, CA, United States

**Keywords:** HIV, sexual health and wellness, African American women, black church, HIV prevention, depression

## Abstract

This report describes the recruitment of a sample of older African American women to test the effectiveness of an educational HIV prevention intervention that sought to reduce depressive symptoms and thereby HIV risks in this population. The outreach venue is the Black church. A framework for maximizing response is suggested. Of 62 women who participated in two arms of the intervention, 29 were assigned randomly to a four-session discussion group (experimental condition) and 33 were assigned to a one-session informational group (control condition) focused on HIV prevention education. Between-within subjects analyses of variance showed that participation in the study was associated with a significant improvement in the women's psychological status, i.e., decreased depressive symptoms. This change in depressive symptoms was due in part to the experimental condition assignment. Implications for future HIV prevention interventions, research, and methods used to maximize the probability of response among older African American women are discussed.

## Introduction

Depression is common among older African American women (1–4). In addition, HIV is a prevalent public health issue whereas older African American women are disproportionately burdened (5–9). Depressed mood is associated with increased sexual risk-taking behavior (10), depression has also been identified as an HIV risk factor (7, 9). Yet, few HIV prevention interventions have focused on older African American women at the intersection of depression and HIV (7).

HIV prevention programming is of particular importance for older African American women who live in resource-constrained communities because important scientific questions concerning how to address HIV prevalence and prevention among vulnerable women in this population can only be addressed effectively if older African American women are included in intervention trials in ample numbers. HIV intervention initiatives seldom have optimal levels of participation among older African American women because of misconceptions that HIV only affects young people (11), even though older adults have many of the same risks and transmission vulnerabilities for HIV infection as younger groups (5, 9, 12). Research has shown that behavioral interventions can curtail HIV risks among adult women (13). However, enrollment of racial/ethnic women into clinical research trials remains a substantial barrier to conducting ethnically representative research (14). The question is, how might researchers and service providers reach and recruit a population of older African American women to conduct representative research focused on the delivery of HIV education and prevention programming?

This report describes the recruitment of a sample of older African American women to test the effectiveness of an HIV education intervention that sought to reduce depressive symptoms and thereby HIV risks in this population. The outreach venue of interest is the Black church for several reasons: Pew Research Center indicates that nearly six-in-ten older African American women acknowledge their participation in church services at least once a week (15, 16). Additionally, the Centers for Disease Control and Prevention (CDC), is a strong advocate for the inclusion of faith leaders and the church in HIV prevention endeavors in the Black community (17–19).

## Conceptual framework for sample recruitment

African American churches have been defined as religious organizations, “whose decision-making is controlled by African American individuals” (20). Often these churches spearhead community activities and may be the only organizations in the African American community headed and controlled by African Americans (21–23). They often serve as implementers in health promotion programming for church members who may be less likely to engage in health and wellness initiatives within the professional service arena (22–24). As such, some have described the church as a means of addressing sociocultural barriers to recruitment of minority groups to clinical trials (21, 25). Others describe the Black church as a likely site for the provision of services to the Black elderly (26, 27).

Swanson and Ward (25) identify sociocultural barriers as trepidation and wariness or mistrust of funded research, the scientists conducting the research, and/or the institutions at which the research is conducted. The Tuskegee Syphilis Study is a foundational example of research that has contributed to African Americans’ mistrust of research (28). Some attribute this mistrust also to perceptions of contemporary systemic racism (29, 30). Thus, an important first step in the recruitment of vulnerable older African American women to a pilot study involving the delivery and testing of an HIV prevention program in a local African American church involves gaining the trust of the church leaders whose input and endorsement are invaluable in providing access to potential participants.

Theoretically, Bronfenbrenner and Ceci's (31) ecological perspective provides a framework for engaging a church and recruiting a sample of older women. Although in this perspective, the ecological environment is divided into a set of five different systems—the microsystem, the mesosystem, the exosystem, the macrosystem, and the chronosystem—our interest at the outset was in the influences of the microsystem in the church. We considered this the most immediate environmental setting that would influence our ability to recruit a sample. In the church, the microsystem consists of relations between and among older women and significant others, including the pastor, church leaders, and other church members. Some have described these relations as a “kinship network” of importance within some Black churches (32). Thus, we started out by addressing possible sociocultural barriers first through contact with the pastor, whom we considered the church leader.

In the present study, sociocultural barriers were addressed in the following manner. The first contact consisted of a telephone call to the head pastor of the church. Since some have found that having a similar racial background to possible study participants can sometimes, if not always, facilitate a greater sense of trust (21, 25, 33), the first author/principal investigator informed the pastor of her race (African American) and the aim of the proposed study; i.e., to deliver an HIV prevention intervention to older African American women who are at high risk for HIV (5–9). The focus of the discussion with the pastor during the telephone contact was on the vulnerability of older African American women to HIV infection, their lack of adequate knowledge about the relevant risk factors, the paucity of HIV intervention programs that focus on this population, and possible benefits for the church in helping to fill an important gap in data and methods for public health services (25). What followed, as expected, was an invitation to meet face-to-face and, subsequently, several meetings with church leaders.

We reasoned that if our interactions with the church leaders resulted in a high regard for the aims of the research, our efforts to recruit participants would be successful. In several meetings with key stakeholders in the church, the evidence on HIV risks for older African American women and the unique position of the church in engaging this group were reviewed. Specifically, our objective was to exhibit trustworthiness, knowledge of the subject matter, concern for the population of interest, and a desire to get their help in incorporating the congregants’ beliefs and lifestyles into materials for marketing and educational programming (25, 34). These discussions resulted in an invitation to attend multiple weekly programs at the church in August, September, and October 2018, as well as several Sunday services, to describe the clinical trial to church members and solicit participants. The study was described as an HIV education program focused on older African American women; a population affected disproportionately by the HIV epidemic. Prospective participants also were informed that we were aware that some of them might already be knowledgeable about HIV and, if so, we were interested in learning from them how best to present this information to their less knowledgeable cohorts. This approach was intended to convey our view of them, as people that could be contributors in meeting the objectives of this initiative, not just people in need of help. This also was an example of what some have described as a “consulting, collaborative, respectful approach to sample recruitment” (7, 34).

## Procedure and sample

Women who agreed to participate in the study were given an appointment to attend a group meeting, during which the study was described again and written, informed consent was obtained (7). The criteria for inclusion were race/ethnicity (African American/Black), gender (female), and age (aged ≥50 years). Of the women screened, 62 who were eligible participated in the study. During this meeting, the women were assigned randomly to the experimental condition (*n* = 29) or the control condition (*n* = 33), using the sample function in the R statistical language (35). Then, participants completed a questionnaire measuring their pre-intervention depressive symptoms (7).

Women assigned to the experimental arm of the intervention returned the next week to start the 4-week HIV program focused on HIV prevention. Those assigned to the control group returned on a different day for participation in the one-session control group arm of the intervention. The retention rate from pretest to 6 weeks posttest was 95.2 percent.

The curriculum for both arms of the intervention, informed both by Bronfenbrenner and Ceci's (31) ecological perspective and Bandura's (36) social cognitive theory, included knowledge about safer-sex negotiations, discussions with intimate partners about decisions related to sexual health, empowerment and self-advocacy, the deconstruction of myths, and the importance of HIV testing (7). The four-session experimental group also included opportunities for the women to acquire knowledge from group members *via* examination, replication, and demonstration in the context of social interactions and experiences (7, 36). Here, our focus is on the intersection of depression and HIV. Thus, we present a test of the effect of both arms of the intervention on depressive symptoms in the analyses that follow. The analyses and results that follow have been previously published elsewhere (7). They are included here to show that the intervention yielded promising results under controlled conditions. This report differs from our previous work as it contributes to the field of HIV research describing in greater detail the methodological approach and framework used to recruit an underrepresented group consisting of African American older women for an HIV prevention trial. The current report presents a strategy for maximizing the probability of response as well. This is important because, as indicated earlier, older African American women are rarely the focus of HIV prevention research. This report builds on our previous work that focuses on ways to deliver cost-effective interventions to older African American women who are not HIV positive and who lack adequate knowledge about transmission risks (7).

## Measures

### Depressive symptoms

Depressive symptomology was assessed using the Center for Epidemiologic Studies Depression Scale (CES-D) at baseline and 6-week follow-up. The standard 16+ cutoff was applied to define case-level symptomatology (37). The *CES-D* is a 20-item self-report scale designed to measure depressive symptomatology in the general population. The items on the scale are symptoms associated with depression, such as diminished appetite, restless/interrupted sleep, or feelings of loneliness, that have been used in previously validated longer scales (37, 38). Response options ranged from Less than 1 day to 5–7 days in the past week. Cronbach's alpha was.81 at time 1 and.84 at time 2.

### Demographics

Educational attainment was measured on a seven-point scale (1 = grade school to 7 = BA/BS Degree) that asked participants for the highest level of education completed. To determine employment status, participants were asked if they were currently employed at time 1 and time 2. Annual income was indicated on a scale ranging from less than $5,000 to more than $100,000 in the past 12 months. Two variables (coded 1 if yes and 0 if no) were constructed to designate relationship status, i.e., the women were asked if they were currently in a romantic relationship and if they were in a sexual relationship. Marital status was indicated on a seven-point scale (1 = never married to 7 = domestic partnership).

## Analytic strategy

Data from the pretest and posttest questionnaire were entered in SPSS version 25. An independent-measures *t* test and a repeated measures analysis of variance with a between-groups factor (control vs. experimental conditions) were conducted to evaluate whether the interventions had an effect on depressive symptoms. The assumptions of normality and homogeneity of variance were assessed and met. Specifically, the between-groups main effect examined whether there was a change in depressive symptoms among the women in the experimental group and those in the control group at the 6-week post-intervention follow-up. The repeated measures main effect examined whether there was variability in depressive symptoms from pretest to posttest and, if so, the interaction effect examined whether the difference from pretest to posttest varied as a function of group membership (see, also, 7).

## Results

The final sample consisted of 62 women. The women were on average 68.32 years old (SD = 8.43, range = 50–89). Most (76%) had some education beyond high school and were no longer employed (73%). Fifty-three percent of the women were divorced, and close to a fourth (24%) were involved in a romantic relationship that was sexual.

Mean scores for depressive symptoms at time 1 were 8.56 (SD = 7.09) for women in the experimental group and 8.31 (SD = 6.68) for those in the control group. Results of an independent-measures *t* test revealed no significant difference between the two groups at time 1 on the CES-D (*t* = −.133, df = 52, *p* = .90). There was a change in depression scores from time 1 to time 2. This change was greater for women who participated in the four-session experimental group, as opposed to those who participated in the one-session control condition. The repeated measures analysis of variance with a between-factor (control condition vs. experimental condition) showed a significant interaction between time and experimental condition, *Wilk's Lambda* = .942, *F* (1,49) = 3.024, *p* = .088, indicating that the change in depressive symptoms partly depended on the experimental condition assignment.

**Figure 1:**
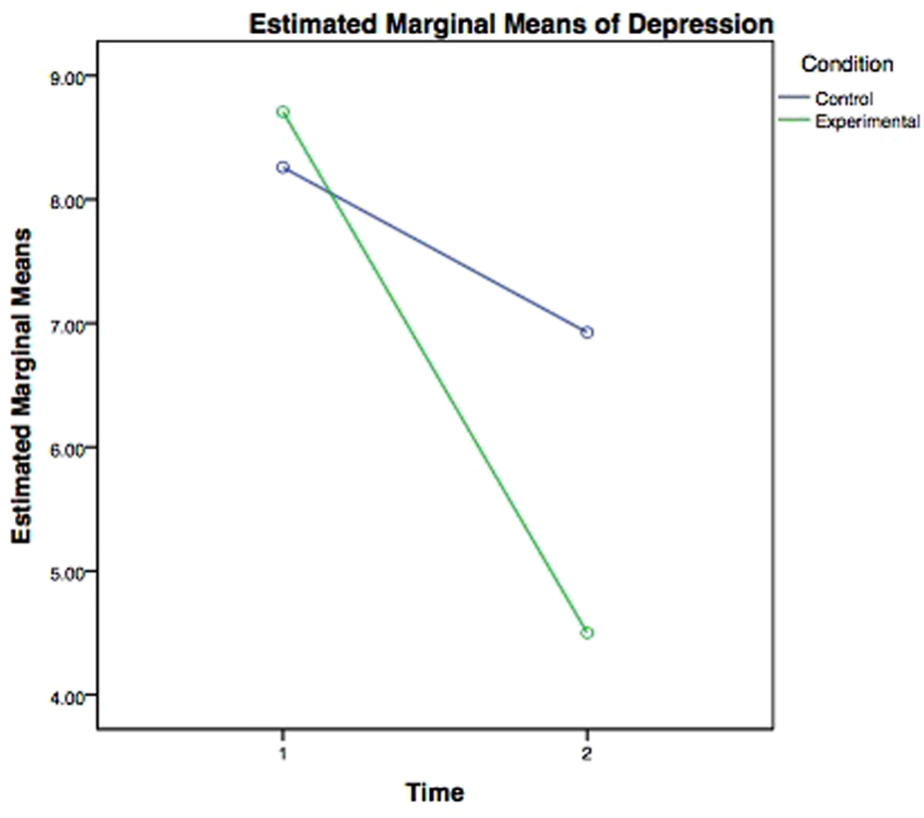
Estimated marginal means of depression for participants across time by condition.

**Figure 2:**
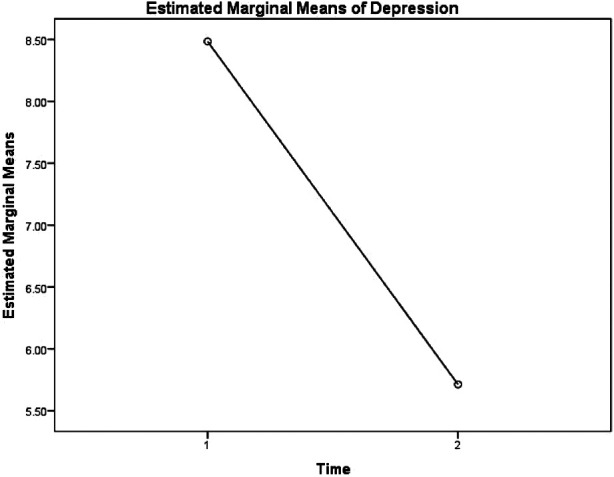
Estimated marginal means of depression for participants averaged over condition.

Results of the interaction between time and experimental condition are displayed in Figure 1. Explicitly, simple effects tests revealed a significant decrease in depression scores for participants in the experimental condition from time 1 (M = 8.71, SE = 1.44) to time 2 (M = 4.50, SE = .89), *Wilk's Lambda* = .800, *F* (1,49) = 12.237, *p* = .001. Conversely, the simple effects tests showed no significant change in depression scores for participants in the control condition from time 1 (M = 8.26, SE = 1.35) to time 2 (M = 6.93, SE = .84), *Wilk's Lambda* = .973, *F* (1,49) = 1.38, *p* = .245. These findings show that the experimental intervention was successful in decreasing depression scores from time 1 to time 2. Additionally, the results showed a marginally significant repeated measures main effect, *Wilk's Lambda* = .813, *F* (1,49) = 11.234, *p* = .002, indicating a difference in depressive symptoms among all participants from time 1 to time 2, averaged over condition. Specifically, the mean score for depressive symptoms for all participants was 8.48 (SE = .99) at time 1 and decreased to 5.71 (SE = .61) at time 2. The results of the repeated measures main effect are displayed in Figure 2.

## Discussion

This study examined how researchers and service providers might reach and recruit a population of older African American women to conduct representative research focused on the delivery of HIV education and wellness services in a church setting. We tested the effectiveness of an educational HIV prevention intervention that sought to reduce depressive symptoms and thereby HIV risks in this population. We anticipated successful recruitment of older African American women through accessing the microsystemic relationships within the church community. We treated these practices as vital prevailing elements in HIV prevention strategies for older African American women. An intervention with a pretest/posttest experimental design was carried out. The findings indicate that while all participants in the study showed an improvement in depressive symptoms, those who participated in the four-session experimental group program showed a significantly greater improvement. This suggests promise for HIV interventions that go beyond informational content for older African American women who may be at risk for depression that ranks high among HIV risk factors.

While further testing is necessary, if effective, our results suggest that an HIV education intervention with older African American women in a church setting may yield positive outcomes resulting from discourse in a communal environment about their relationships with the intimate partners in their lives. Others also have found that programming that includes interpersonal communication with existing social networks, as reflected in the current study, can be beneficial for older African American women (38, 39). If such programs also are associated with reduced depression, as our findings suggest, then this is an important finding for HIV prevention work, given studies showing that depressed mood is associated with increased sexual risk-taking behavior (10).

Our findings also are consistent with other research indicating the significance of communities of faith as collaborators in health promotion and education programming for populations that may be challenging to engage (40, 41). For example, the content embedded in the 4-sessions were modeled after ancient religious literature based upon a well-known story to a wise person building a house upon a rock. Akin to this analogy, sessions 1–4 were designed to signify the building blocks of HIV prevention messaging based on each phase of assembling a home (e.g., Session 1, Foundation; Session 2, Framing; Session 3, Roofing; Session 4, Finishing) (Table 1). In addition to the spiritual symbolism infused in the structure of the intervention, the women enrolled in the intervention group were invited to collaboratively establish group rules. During this time group members were able to create agreed upon guidelines whereby they would interact with one another and as a community. One of the first established rituals included incorporating prayer at the beginning and closing of each session. The women also agreed to maintain the discussions in confidence among group members, thereby deepening cohesion and trust, which seemed, in turn, to enhance their investment in the group process and the importance of modeling behavior consistent with HIV prevention, i.e., they seemed to enjoy relating to and learning from each other's experiences. These considerations may have contributed to positive mental health outcomes, exemplified by the change in depressive symptoms from time 1 to time 2. Some have conjectured that such outcomes are possibly precursors of women's self-assurance, modeling behaviors, and accountability (43). This may explain the connection between positive mental health outcomes and HIV prevention, which is also consistent with social cognitive theory (36).

**Table 1:** Curriculum outline: even me women's health circle (EMWHC).

Session no/Title	Components
(1) Foundation: Cultural and Historical Background	Introduction and Overview Getting to know you/Icebreaker activity Establishing group rules Historical effects of shared trauma (SHM) Addressing stigma experientially (relatable vignettes and discussion questions) (SHM) Homework (SHM)^a^ Closing, positive affirmation, prayer (SHM)^a^
(2) Framing: What do You Know About HIV?	Group Prayer^b^ Opening and welcome^a^ Review last session^b^ Review Homework^b^ HIV modes of transmission activity Women's risk assessment test (HIV perceived risk) Defining stigma (brainstorming exercise) Stigmatization process model (how stigma impacts individuals and the community) Results of Women's risk assessment test Discussion (Q&A)
(3) Roofing: Even Me (Observational Learning)	Film Introduction Viewing of Documentary film (HIV content) Discussion (Q&A) Closing (positive affirmations)
(4) Finishing: HIV & Aging	Differentiating symptoms of HIV vs. aging Dating (exploring new relationships) Safer sex practice/negotiation Discussing sexual health with health care providers Role-plays and modeling exercises (SHM) Discussion (Q&A) Review/Resources

(SHM) Elements incorporated from the sexual health model (7).

^a^ These elements were present in sessions 1–4.

^b^ These elements were present in sessions 2–4.

Previous studies have reported positive connections among “spirituality/religion, social support, and promotion of health-related behaviors among African American women” (42–44). All these elements were integrated into the 4-session curricula. One example worth noting is the short health communication film—Even Me (6), one of the first health communication films developed with and for racial or ethnic minority older people to increase awareness and encourage protective behavioral change related to HIV—was watched and discussed during session 3 (see Table 1). The incorporation of this film was critical in the development of the curriculum as it included relatable members of the community sharing their experiences of living with HIV and is centered on one of the core concepts of social cognitive theory: observational learning/modeling (36).

There are, however, several limitations that should be acknowledged. First, the sample was small, and the study employed a non-probability convenience sampling technique. Further research with additional and larger samples is needed to explore more wholly the issues of focus here. For example, it is important to test more rigorously whether health and education interventions that aim to enhance HIV knowledge acquisition and HIV prevention among older African American women can be carried out in Black churches in resource-constrained communities on a large scale. In addition, examining whether such interventions might be associated with reduced depressive symptoms, safer sexual behaviors, and a greater likelihood of regular HIV testing among otherwise healthy older African American women is important. Given that random assignment in the present study did not guarantee that the experimental and control groups were matched or equivalent, if similar findings are generated in additional studies, the validity of the present results will be supported.

Finally, it is important to consider that the Black church is diverse and should not be viewed as a monolith. Black churches vary in denomination, theological traditions, size, geographical region, and congregational make-up. These factors should be well-thought-out when conducting research in this setting. To this end, it is important to keep in mind that both the approach and protocols in gaining access to faith leaders and congregants will also vary. While our procedures were successful in gaining access to faith leaders, recruiting and retaining older African American women to participate in this programming, the steps outlined in this study should be considered a starting point for further testing and replication. Nevertheless, this study does begin to close an important gap in current evidence on HIV sexual health and wellness prevention interventions focused on older African American women, an under-studied population. Our strategies for engaging this population suggest that successful collaborative efforts between and among researchers, social service practitioners and faith-based organizations are possible.

## Data Availability

The raw data supporting the conclusions of this article will be made available by the authors, without undue reservation.
